# A Heat-Induced Mutation on VP1 of Foot-and-Mouth Disease Virus Serotype O Enhanced Capsid Stability and Immunogenicity

**DOI:** 10.1128/JVI.00177-21

**Published:** 2021-07-26

**Authors:** Hu Dong, Yuanlu Lu, Yun Zhang, Suyu Mu, Nan Wang, Ping Du, Xiaoying Zhi, Xiaobo Wen, Xiangxi Wang, Shiqi Sun, Yanming Zhang, Huichen Guo

**Affiliations:** a State Key Laboratory of Veterinary Etiological Biology, National Foot and Mouth Disease Reference Laboratory, Lanzhou Veterinary Research Institute, Chinese Academy of Agricultural Sciences, Lanzhou, Gansu, China; b College of Veterinary Medicine, Northwest A & F University, Yangling, Shaanxi, China; c National Laboratory of Macromolecules, Institute of Biophysics, Chinese Academy of Science, Beijing, China; d College of Animal Science and Technology, Hainan University, Hainan Key Lab of Tropical Animal Reproduction and Breeding and Epidemic Disease Research, Haidian Island, Haikou, China; e College of Animal Science, Yangtze University, Jingzhou District, Jingzhou, People’s Republic of China; University of Kentucky College of Medicine

**Keywords:** foot-and-mouth disease virus, thermo-resistance, amino acid mutation, immunogenicity, three-dimensional structure, virus-like particles

## Abstract

Foot-and-mouth disease (FMD) is a highly contagious viral disease affecting cloven-hoofed animals that causes a significant economic burden globally. Vaccination is the most effective FMD control strategy. However, FMD virus (FMDV) particles are prone to dissociate when appropriate physical or chemical conditions are unavailable, such as an incomplete cold chain. Such degraded vaccines result in compromised herd vaccination. Therefore, thermostable FMD particles are needed for use in vaccines. This study generated thermostable FMDV mutants (M3 and M10) by serial passages at high temperature, subsequent amplification, and purification. Both mutants contained an alanine-to-threonine mutation at position 13 in VP1 (A1013T), although M3 contained 3 additional mutations. The selected mutants showed improved stability and immunogenicity in neutralizing antibody titers, compared with the wild-type (wt) virus. The sequencing analysis and cryo-electron microscopy showed that the mutation of alanine to threonine at the 13th amino acid in the VP1 protein (A1013T) is critical for the capsid stability of FMDV. Virus-like particles containing A1013T (VLP_A1013T_) also showed significantly improved stability to heat treatment. This study demonstrated that Thr at the 13th amino acid of VP1 could stabilize the capsid of FMDV. Our findings will facilitate the development of a stable vaccine against FMDV serotype O.

**IMPORTANCE** Foot-and-mouth disease (FMD) serotype O is one of the global epidemic serotypes and causes significant economic loss. Vaccination plays a key role in the prevention and control of FMD. However, the success of vaccination mainly depends on the quality of the vaccine. Here, the thermostable FMD virus (FMDV) mutants (M3 and M10) were selected through thermal screening at high temperatures with improved stability and immunogenicity compared with the wild-type virus. The results of multisequence alignment and cryo-electron microscopy (cryo-EM) analysis showed that the Thr substitution at the 13th amino acid in the VP1 protein is critical for the capsid stability of FMDV. For thermolabile type O FMDV, this major discovery will aid the development of its thermostable vaccine.

## INTRODUCTION

Foot-and-mouth disease (FMD) is a global disease of cloven-hoofed animals, highly infectious, and antigenically variable, which poses a major threat to the animal industry and causes enormous economic losses (1, 2). Effective vaccination combined with stringent control programs have contributed to controlling the epidemic in some countries (3) and even successful eradication in Europe, North America, and some countries in Asia (4, 5). However, FMD emerges and reemerges in some countries where the disease has been well controlled or eradicated, due to rapid genetic variation of the virus and vaccine failure (6–8).

FMD virus (FMDV) is a member of the *Aphthovirus* genus within the *Picornaviridae* family. It is approximately 30 nm in diameter, forms an icosahedral structure with a sedimentation coefficient of 146S, and consists of 60 copies of each of the capsid proteins VP1 to VP4 (9). FMDV also forms some intermediates during replication, such as 75S empty capsids, 12S pentamers, or 5S protomers (10, 11). The capsid protein precursor (P1 + 2A) of picornavirus is initially digested by 3C protease into VP0, VP1, and VP3, which form a protomer. Five protomers assemble into a pentamer and 12 pentamers generate an icosahedral symmetric empty capsid (RNA-free capsid). Mature virions are ultimately formed, with the genome’s entry into capsids and VP0 autocatalytic cleavage into VP2 and VP4 (12). FMDV is more highly sensitive to acids and heat than other picornaviruses (10), which is conducive to virus infection and replication (13, 14). During the normal virus purification process, nearly 10% of the intact particles (146S) dissociates into 12S pentamers (15). Empty capsids, which lack an interaction between RNA and proteins, are less stable than 146S particles (16). Doel et al. previously confirmed by experiment that the protective immunity of FMDV is mainly dependent on intact (146S) and empty (75S) particles, while the dissociated 12S pentamers contribute little to protective immunity of FMD vaccines, and the amount of neutralizing antibody induced by the latter is very low (10, 17, 18). The dissociation of particles due to thermoinstability can lead to poor efficacy of vaccines and a short duration of immunity. Vaccine efficacy correlates with the content of intact particles in the vaccine formulation (19). Therefore, the stability of the FMD vaccine will contribute to their potency, especially for inactivated virions and virus-like particles (VLPs). Nowadays, VLPs with good representation of the antigenic conformation of authentic virus can be prepared by various recombinant expression systems (20). Due to their gene-free nature, VLPs do not pose risks of virus escape during industrial preparation of infectious virus, as occurs with conventional vaccines. However, similar to natural (75S) empty capsids, VLPs have a lower thermostability than 146S particles. Hence, VLPs with improved thermal stability are the most promising novel FMD vaccine candidates.

In 2003, Mateo et al. (21) first improved the capsid thermal stability of FMDV by amino acid modification based on the three-dimensional structure of FMDV. Afterward, a series of studies focused on the modification of amino acids at the interpentamer interface to prevent virus capsid dissociation, including strategies of reducing electrostatic repulsion (22–25) or introducing covalent (26) and conjugate bonds (15, 26, 27). Great progress has been made in improving the stability of FMDV serotypes A, Asia 1, and SAT2, including empty capsids and inactivated virions, through thermodynamic calculation. In this study, we focused on improving the thermostability of a type O virus that is less stable than other FMDV serotypes (10). Heat-resistant viruses were selected under stress and further analyzed for capsid stability and immunogenicity. Cryo-electron microscopy (cryo-EM) analysis of the stabilized capsid was done to demonstrate the underlying molecular mechanism for stabilization.

## RESULTS

### Selection of thermostable FMDV strains.

**FIG 1:**
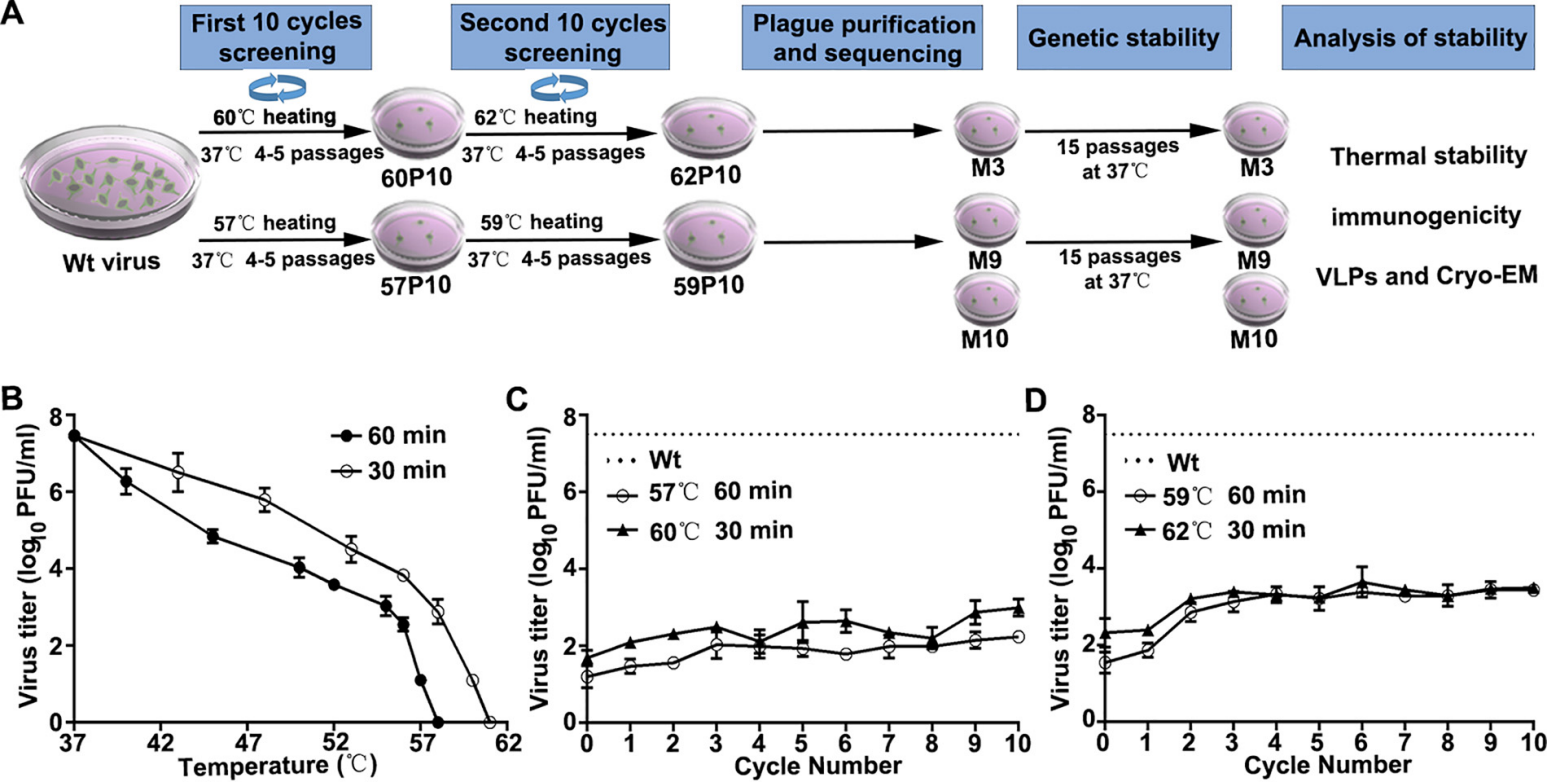
Selection of FMDV thermostable mutants. (A) The flowchart of screening heat-resistant FMDV. (B) The titer of virus incubated at different temperatures was determined by plaque assay, samples were repeated in duplicate, and data are presented as the mean ± SD. (C) The virus was heat treated at 57°C for 60 min or 60°C for 30 min, followed by subpassaging at 37°C until the virus titers were restored; virus pools were named 57P01 and 60P01, respectively. There were 10 repeated cycles of thermal selection and passage. (D) Viruses (57P10 and 60P10) were thermally selected further with the selection pressure increased to 59°C for 60 min or 62°C for 30 min according to the above method. The dashed line represents wt virus grown at 37°C without heat treatment, and the virus titers were all determined by plaque assay. Data are presented as the mean ± SD. Experiments were repeated in duplicate.

**FIG 2:**
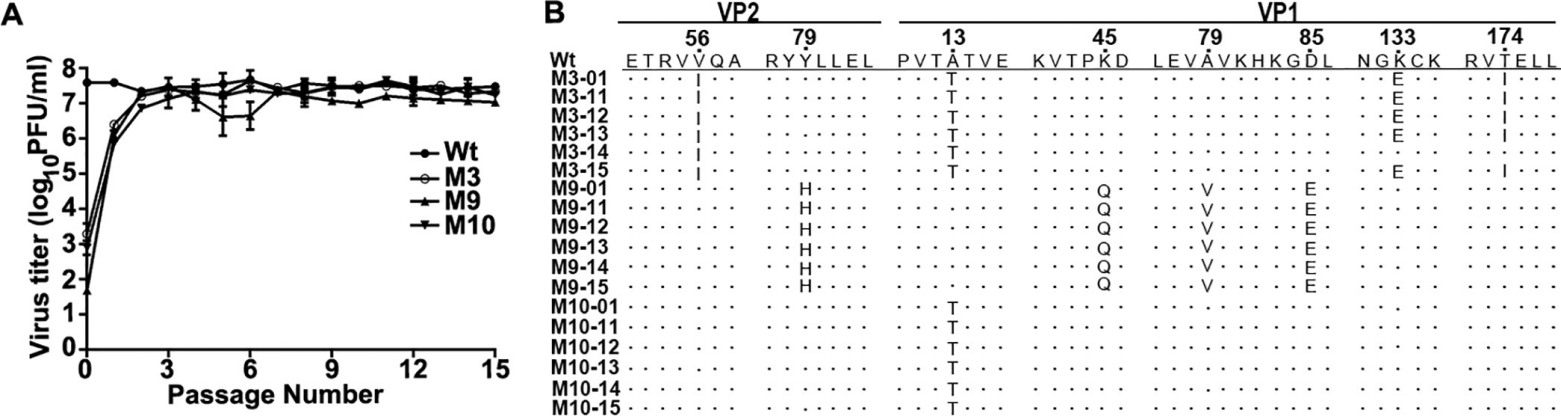
Genetic stability of thermostable FMDV variants. (A) The dynamics of the virus titer of selected mutants during 15 passage cycles, samples were repeated in duplicate, and data are presented as the mean ± SD. (B) Sequence alignment of the relevant parts of the P1 region of mutants M3, M9, and M10 where amino acid changes occur compared with the wild-type virus. Mutant viruses from passage 1 and passages 11 to 15 were subjected to sequence analysis to show the stability of the mutations.

A complex process of several steps was used in this study to select heat-resistant FMDV mutants (Fig. 1A). We first demonstrated that the wild-type (wt) virus was completely inactivated after incubation at 58°C for 1 h or 61°C for 30 min (Fig. 1B). At 1°C below these temperatures, virus titers were 10,000-fold decreased to 10^1.2^ PFU/ml (at 57°C for 1 h) or 10^1.69^ PFU/ml (at 60°C for 30 min) (Fig. 1C), and the survival virus pools were named 57P01 and 60P01, respectively, which were continuously passaged at 37°C until the virus titer returned. After 10 repeated cycles of thermal selection, heating at 57°C or 60°C and then passaging at 37°C, the titer increased to 10^2.24^ PFU/ml (57P10) and 10^3.0^ PFU/ml (60P10). The virus populations 57P10 and 60P10 were subjected to another 10 cycles of thermal selection, heating at 59°C and 62°C, respectively, and followed by passage at 37°C. The resultant virus titers increased to 10^3.44^ PFU/ml (59P10) and 10^3.5^ PFU/ml (62P10) (Fig. 1D). Finally, plaque purification of the virus pools 59P10 and 62P10 yielded 12 thermostable mutants named M1 to M12. Sequence analysis of the P1 region revealed the following three unique mutants with stable nonsynonymous capsid mutations compared with the wild-type virus: M3 (V2056I, A1013T, K1133E, and T1174I), M9 (Y2079H, K1045Q, A1079V, and D1085E), and M10 (A1013T). Notably, mutant M10 contained only a single amino acid mutation that was also present in mutant M3. These three mutants were passaged for 15 cycles in the absence of heat stress in order to obtain stable viruses. Their titers restored to the level of preheat treatment after the fourth passage (Fig. 2A) without any compensatory mutation occurring during further passage (Fig. 2B).

### Heat resistance of mutants M3, M9, and M10.

**FIG 3:**
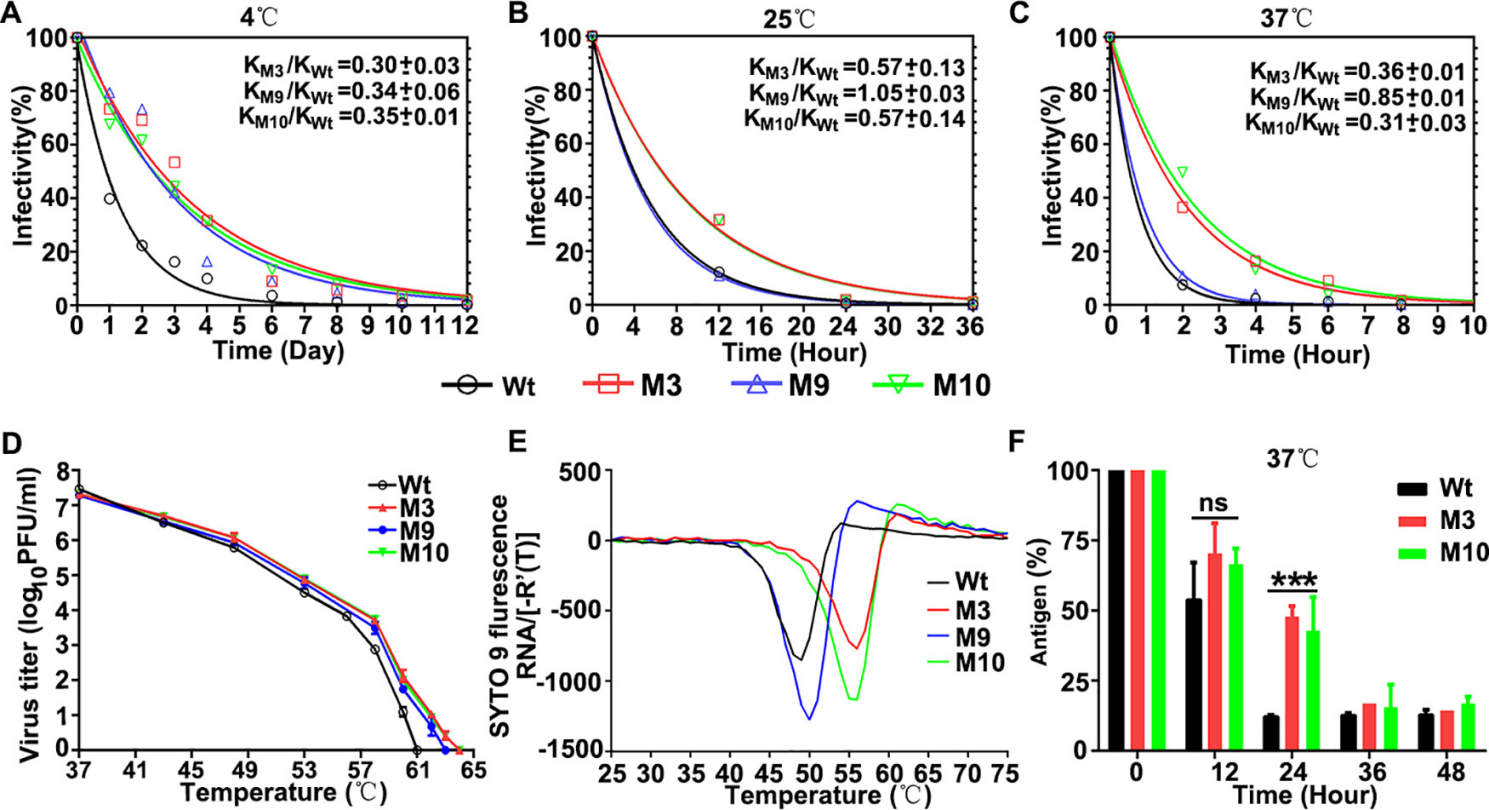
Inactivation kinetics and heat resistance of FMDV serotype O mutants. The percentages of remaining infectious particles of wild-type (wt) virus and mutants M3, M9, and M10 were determined following heat treatment at 4°C for 12 days (A), 25°C for 36 h (B), or 37°C for 10 h (C), and the inactivation rate constants were calculated. (D) The titers of virus incubated at different temperatures were determined by plaque assay. Compared with the wild-type virus, the temperature for complete inactivation of mutants M3, M9, and M10 increased to 64, 63, and 64°C, respectively. Data are presented as the mean ± SD. (E) RNA release from capsid as detected by SYTO 9 fluorescence in PaSTRy assay. The complete RNA release temperature of the wt virus was 49.0°C, M3 was 56.0°C, M9 was 50.0°C, M10 was 56.0°C. (F) Double antibody sandwich ELISA to detect the remaining intact antigens after heat treatment at 37°C. The percentage of intact particle of wt virus and mutants are statistically different (*n* = 2; ± SD; ns, not significant; ***, *P < *0.001; **, *P < *0.01).

The thermal stability of the mutants was verified by measuring their infectivity at 4, 25, and 37°C. The mutants M3 and M10 had a lower inactivation rate than the wild-type virus at all 3 temperatures. Of note, mutants M3 and M10 had similar inactivation rates that were considerably lower than those of M9 and wild-type virus. The time required for inactivating 50% particles of the selected strains (M3 and M10) was significantly prolonged. At 25 and 37°C, the percentage of residual infectious particles of M3 and M10 mutants was significantly higher than that of wild-type virus and M9 mutants (Fig. 3A to C). Also, the temperature required for complete inactivation of viral infectivity of mutants M3 and M10 increased to 64°C and mutant M9 to 63°C, compared with 61°C for the wild-type virus (Fig. 3D).

The thermal stability was further analyzed using a particle stability thermal release assay (PaSTRy), where the temperature resulting in the release of the RNA genome (Tr) is determined by SYTO 9 fluorescence. The capsids of the mutants M3 and M10 were able to resist higher temperatures than that of the wild-type virus (Fig. 3E). Among these three mutants, the Tr values of M3 and M10 mutants were increased to the largest extent of 6 to 7°C compared with that of the wild-type strain. The remaining integrity of antigen content after continuous heating treatment at 37°C was detected by a double antibody sandwich enzyme-linked immunosorbent assay (ELISA) (28), which can specifically detect intact FMDV particles using llama single-domain antibody M170. The results showed that a 50% mutant virus that can maintain the integrity of viral particles for up to 24 h at 37°C, while the wild-type virus was almost completely dissociated (Fig. 3F).

### Immunogenicity of mutant particles.

**FIG 4:**
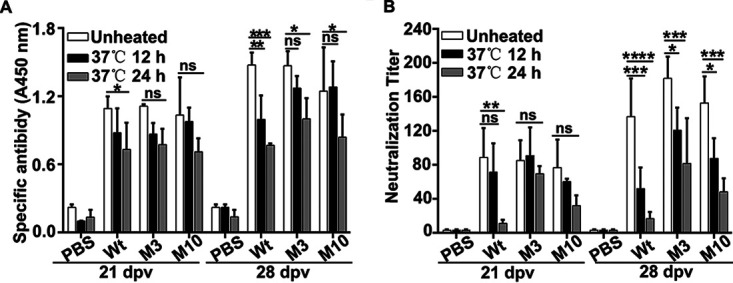
Comparison of antibody titers induced by wild-type virus and mutants M3 and M10. Guinea pigs were immunized with inactivated viruses that were either unheated or heated at 37°C for 12 h or 24 h. Serum samples were collected at 7, 14, 21, and 28 days postvaccination (dpv) to detect the antibody titers by ELISA (A) or VNT (B). The level of unheated and heated samples was statistically different (*n* = 6; ± SD; ****, *P < *0.0001; ***, *P < *0.001; **, *P < *0.01; *, *P < *0.05).

The immunogenicity of mutants M3 and M10 and wild-type virus was assessed by vaccination of guinea pigs both after 12 h or 24 h of incubation at 37°C and without prior heat treatment. Antibody responses were detected by both ELISA (Fig. 4A) and virus-neutralizing test (VNT) (Fig. 4B). The wild-type virus was highly sensitive to heat treatment and showed a highly significant (*P* < 0.001 or *P* < 0.0001) decrease in antibody responses after heat treatment both by ELISA at 21 and 28 days postvaccination (dpv) and VNT (Fig. 4). In contrast, mutant M3 and M10 showed resistance to heat treatment and had a slight reduction in antibody titers compared with the unheated virus only at 28 dpv by ELISA (*P* < 0.05) (Fig. 4A) or VNT (*P* < 0.05 or 0.001) (Fig. 4B). Of note, the heat-treated M3 and M10 viruses elicited significantly higher neutralizing antibody responses (*P* < 0.01) at 28 dpv (Fig. 4B) than that of the wild-type counterpart. All together, these results demonstrated that mutants M3 and M10 show improved immunogenicity in guinea pigs.

Guinea pigs were subsequently challenged with 100 50% median infective doses (100 ID_50_) of FMDV (GenBank accession no. JN998085.1) on the left hind footpad at 28 dpv. As shown in Table 1, the mutants M3 and M10 provided protection against challenge to 83.3% and 66.7% (for 12 h of incubation at 37°C) and 50% and 16.7% (for 24 h of incubation at 37°C) of guinea pigs, respectively, whereas the heat-treated wild-type virus protected only 33.3% of guinea pigs.

**TABLE 1:** Protection of guinea pigs against FMDV challenge^*a*^

Guinea pig	Protective degree by treatment									
	PBS	Wt			M3			M10		
		0 h^*b*^	12 h^*c*^	24 h^*d*^	0 h^*b*^	12 h^*c*^	24 h^*d*^	0 h^*b*^	12 h^*c*^	24 h^*d*^
1	None	Partial	None	None	Partial	None	None	Partial	Partial	None
2	None	Full	Partial	None	Full	Full	Partial	partial	Partial	None
3	None	Full	Partial	None	Full	Full	Partial	Full	Full	Partial
4	None	Full	Partial	None	Full	Full	Full	Full	Full	Partial
5	None	Full	Full	Partial	Full	Full	Full	Full	Full	Partial
6	None	Full	Full	Partial	Full	Full	Full	Full	Full	Full
Rate of protection (%)	0	83.3	33.3	0	83.3	83.3	50	66.7	66.7	16.7

a Guinea pigs were subcutaneously and intradermally challenged with 0.2 ml 100 ID_50_ of live virus on left rear foot, isolated, and examined for 7 days. Full protection was defined as absence of lesions; partial protection was scored as lesions restricted on left rear foot; none was lesions on all of rear feet. Rate of protection, number of guinea pigs no lesions on both rear feet/total number of guinea pigs.

b Unheated sample.

c Heated sample (for 12 h at 37°C).

d Heated sample (for 24 h at 37°C).

### Amino acid substitution A1013T on capsid improves VLP thermostability.

**FIG 5:**
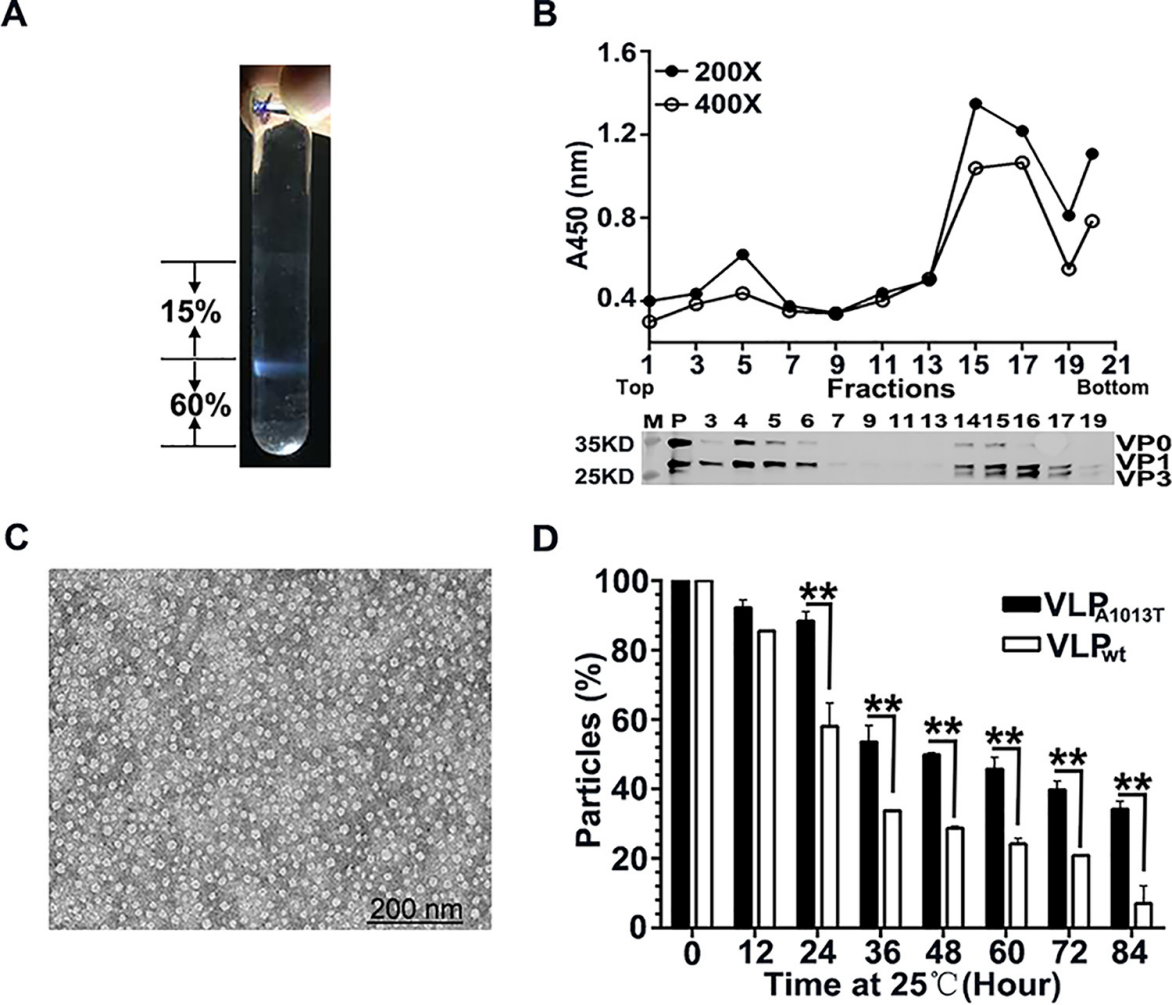
Effect of A1013T mutation on VLP stability. (A) VLP_A1013T_ purification on a 15% to 60% sucrose cushion. (B) VLP_A1013T_s were further purified by a 7.5% to 30% sucrose density gradient, and the diluted samples were analyzed by ELISA and Western blotting using rabbit anti-FMDV polyclonal antibodies. (C) TEM image of VLP_A1013T_. (D) The remaining percentage of VLP_wt_ and VLP_A1013T_ antigens at regular time intervals during incubation at 25°C for up to 84 h were determined by sandwich ELISA. Data are presented as the mean ± SD (*n* = 2). The significance of differences between VLP_wt_ and VLP_A1013T_ is indicated (ns, not significant; **, *P < *0.01).

Mutant M10 contains only the amino acid substitution A1013T which is therefore responsible for its increased thermostability in various assays. To determine whether the mutation A1013T could stabilize VLP capsids, VLPs containing A1013T (VLP_A1013T_) were prepared and highly purified using a sucrose cushion (Fig. 5A) and subsequent sucrose density gradient (SDG) fractionation (Fig. 5B). Analysis of fractions by either the sandwich ELISA or Western blotting identified intact VLPs in fractions 14, 15, 16, and 17 (Fig. 5B), which were pooled and found to contain particles with a diameter between 20 and 30 nm by transmission electron microscopy (TEM) (Fig. 5C). The stability of VLP_A1013T_ was compared to VLP wild type (VLP_wt_) during incubation at 25°C for 84 h by ELISA at regular 12-h time intervals. VLP_A1013T_ showed a higher stability than VLP_wt_ from 24 h until 84 h of incubation at 25°C (Fig. 5D).

### Molecular mechanism of amino acid substitution causing increased capsid stability.

**FIG 6:**
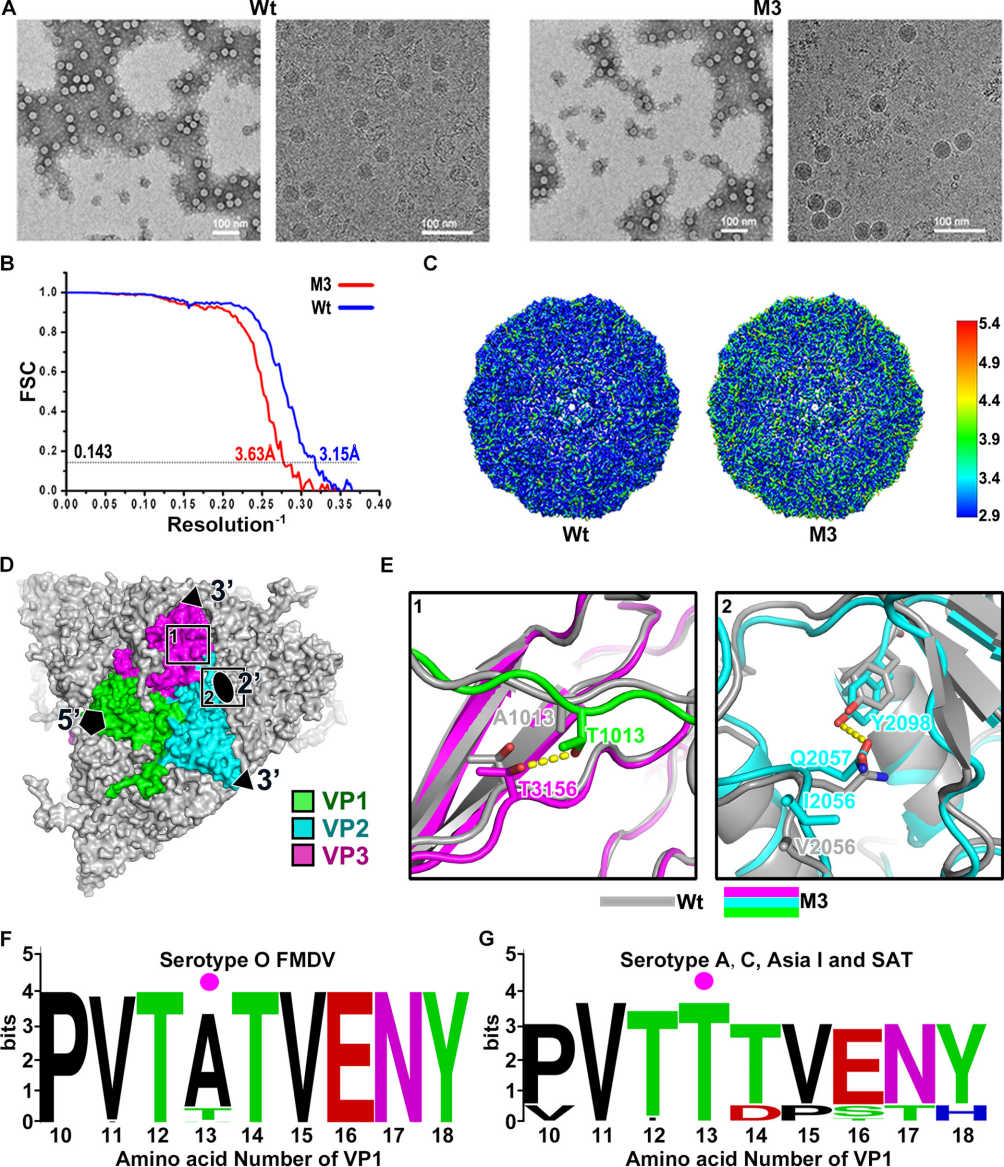
Analysis of amino acid residue interactions. (A) Negative-staining images (left) and cryo-EM images (right) of wild-type virus and mutant M3 particles. (B) Gold-standard Fourier shell correlation (FSC) curves of the final maps. (C) The final maps were analyzed by ResMap, showing a resolution distribution from 2.9 to 5.4 Å of wild-type virus and mutant M3 virus. (D) Distribution of two stabilizing amino acid mutations on the surface of the mutant M3 capsid. The position of the 5-fold (5′), 3-fold (3′), and 2-fold (2′) axis of symmetry is indicated. The VP1, VP2, and VP3 proteins of one protomer are color coded as indicated in the legend, whereas adjacent capsid proteins are indicated in gray. Rectangles 1 and 2 indicate enlargements visible in E. (E) Superimposition of structures of wild-type virus and mutant M3; the wild-type virus is colored gray, and the VP1, VP2, and VP3 proteins of mutant M3 are colored with green, cyan, and magenta, respectively. Hydrogen bonds are shown as yellow sticks. The side chain T1013 on VP1 forms a hydrogen bond with T3156 of an adjacent VP3 protein (E-1). The hydrophilic side chain of Q2057 forms a hydrogen bond with Y2098 of the adjacent VP2 (E-2). (F) Multiple sequence alignment of VP1 protein N terminus from serotype O strains. (G) Multiple sequence alignment of VP1 protein N terminus from FMDV serotypes other than serotype O. The data were shown as WebLogo as described in the Materials and Methods. The 13th amino acid was marked with a magenta dot. Amino acids and their heights indicate the conservation level at each target base position (perfect conservation, 4 bits).

To elucidate the molecular mechanism underlying the stabilization of mutant M3, the structure of the SDG-purified 146S particles of the wild-type and mutant M3 viruses were determined by single-particle cryo-EM (Fig. 6A). Icosahedral reconstruction of particles was achieved at resolutions of 3.1 Å and 3.6 Å, respectively (Fig. 6B and C). The maps for the viral capsid were of sufficient resolution to build and refine the atomic model. Among the four residues mutated in M3 (V2056I, A1013T, K1133E, and T1174I), 3 amino acid residues were well observed, but VP1 residue 133 is located at the flexible G-H loop and could not be well resolved. Mutation T1174I is located near the capsid’s 5-fold axis and, thus, is unlikely to improve capsid stability from a structural point of view. However, mutation A1013T interacts with VP3 from an adjacent protomer within the same pentamer and forms an extra hydrogen bond with T3156 (Fig. 6D), which directly increases mutant M3 stability by increasing the interaction area by about 67 Å^2^ (Fig. 6E, rectangle 1). On the other hand, the bigger hydrophobic side chain of I2056 at the pentamer interface pushes the hydrophilic side chain of Q2057 to form a hydrogen bond with Y2098 of the adjacent VP2, reinforcing the charge interaction between the αB helix and βE-hairpin of VP2 to stabilize the 2-fold axis of the capsid (Fig. 6E, rectangle 2). Three mutated residues in VP1 of mutant M9 (K1045, A1079, and D1085) are located near the 5-fold axis of the capsid. Based on structural biology, these amino acids are unlikely to affect capsid stability. Furthermore, a comparison with other FMD strains shows that amino acids at these positions are highly divergent, suggesting that they reflect random variation. The fourth mutation in M9 Y2079H is therefore most likely responsible for stabilization of M9. Multisequence alignment result showed that alanine (Ala) at 13th amino acid on VP1 of FMDV serotype O is relatively conserved (up to 91%) (Fig. 6F). However, strains from other serotypes most often contain threonine (Thr) at VP1 position 13 (serotypes A, C, Asia 1, and SAT) (Fig. 6G).

## DISCUSSION

As a member of the *Picornavirus* family, FMDV is very sensitive to heat compared with other viruses (10, 29). Improving the heat resistance of the virus capsid is important for the preparation of high-efficiency FMD vaccines. Early studies performed by Bachrach et al. (30) and Mateo et al. (31) indicated the presence of thermostable virus variants in FMDV quasispecies but in small proportions. The genetic variation of the virus will help us to obtain for the dominant virus (32). Whether to break the quasispecies equilibrium under selective pressure, the proportion of heat-resistant population will increased. In this study, a progressive two-step selection method, consisting of 10 cycles of heating selection for each steps, is adopted. The resulting two virus populations were plaque purified to ensure further characterization of clonal populations. Among the three unique mutants found, the mutant M9 displayed the lowest level of stabilization. Mutation Y2079H was most likely involved in stabilization of this mutant. It was previously shown that the virulence of recombinant O PanAsia virus was reduced due to mutation Y2079H, as revealed by the significantly decreased size of plaques (33). A Y2079H amino acid substitution (mutant M9) occurred at the pentameric interface in the βE-hairpin of VP2, which may decrease the efficient electrostatic repulsion force interaction between adjacent pentamers, leading to a prolonged virus replication cycle and decreased virulence. We further focused on another two mutants (M3 and M10) which both contain mutation A1013T and have quite similar thermostabilities. Compared with the wild-type virus, both mutants have an improved thermal stability with lower inactivation rate and their nucleic acid release temperature is improved by 6 to 7°C without affecting virus infectivity. In the absence of capsid-stabilizing agents, the dissociation time of 50% for the mutant viruses or their VLP particles is at least 1 day longer than that of the original ones. Although, the antigens of the wild-type virus and mutants M3 and M10 can induce guinea pigs to produce high specific antibodies after being heated at 37°C for 24 h, but the neutralizing antibody titer produced by the lysed wild-type antigen is significantly reduced (*P* < 0.0001) (Fig. 4B). FMD serotype O is more thermolabile than other serotypes. Mateo et al. performed similar research in the early stage, but they failed to screen out heat-resistant strains. However, this study also confirmed his early speculation that the presence of thermostable virus variants is not a general feature of FMDV quasispecies (31). In addition to quasispecies differences, the type O FMDV used in this study is a long-term cell-adaptive population different from natural FMDV populations.

Similar work has been performed on poliovirus (32, 34), reovirus (35), and other types of FMDV (31); however, to our knowledge, our study represents the first time that researchers have screened out a heat-resistant strain of FMDV type O. The A1013T substitution of mutant strains is highly conserved in other serotypes, except for the O type (Fig. 6F and G). Previous studies had reported that particles of type A, C, and Asia I virions were more heat resistant that type O, especially type A22, which is usually a stable reference strain of FMDV (10). In this study, the dominant strains of FMDV type O were obtained through heat-induced mutation. Thr mutation at the 13th amino acid of VP1 can stabilize the capsid of FMDV. However, it was not feasible to screen the super-heat-resistant virus through genetic variation without affecting its infectivity and fitness. For FMDV, the amino acid at the pentamer interface (2-fold axis) plays a key role in the stability of the capsid, in which amino acid modification can effectively improve the stress resistance of VLPs and virions (15, 22, 26, 27, 36–40), but some amino acid modification will affect virus infectivity and even lethality to the virus. Kotecha et al. used a restrained molecular dynamics strategy to predict a mutation that strengthens the pentamer interfaces, and they applied the results to produce stabilized capsids (15). They confirmed that mutant S93Y of an O1 Manisa strain has a higher dissociation temperature (53.5°C) than the wild-type virus, and more than 50% of the purified S93Y particles remained intact after incubating at 37°C for 12 to 72 h, whereas for the wild type, ∼15% capsids remained whole. The mutation of amino acid 93 from serine (Ser) to tyrosine (Tyr) in VP2 enhanced the stability of the O1 Manisa virus through hydrophobic stacking of aromatic side chains at the 2-fold axis between adjacent pentamers. The amino acid identity of the P1 region of the O/BY/CHA/2010 and O1 Manisa strain is as high as 94%, and the A1013 and S2093 amino acids are conserved in these two strains. Combining findings of this study with the experimental results of Kotecha et al., we speculate that the synergistic effect of A1013T and S2093Y amino acid modification will significantly improve the thermal stability of the O-type FMDV capsid.

In conclusion, in this study, the heat-resistant dominant strains were screened out from O-type FMDV quasispecies by serial passages at high temperature, subsequent amplification, and purification. Mutants showed improved stability and immunogenicity compared with the wild-type virus. An analysis of the mechanism of stable mutants by cryo-EM confirmed that the Thr substitution at the 13th amino acid in the VP1 protein is critical for the capsid stability of FMDV.

## MATERIALS AND METHODS

### Cells and viruses.

Baby hamster kidney-21 (BHK-21) cells were grown in Dulbecco’s modified Eagle’s medium (DMEM; Gibco, CA, USA) supplemented with 100 U/ml penicillin, 100 μg/ml streptomycin, and 10% fetal bovine serum (FBS; Gibco) at 37°C in a CO_2_ incubator. FMDV serotype O strain O/BY/CHA/2010 (GenBank accession no. JN998085.1) was obtained from the OIE/National Foot-and-Mouth Disease Reference Laboratory (Lanzhou, China). The virus titer was determined using a plaque assay as described previously (41). Viruses were incubated at elevated temperatures for inactivation and subsequently at 4°C for 5 min prior to determining virus titer. Virus antigens were purified through a 15% to 45% (wt/vol) sucrose density gradient and examined by negative-stain electron microscopy, as described in our previous study (42).

### Screening and selection of thermostable FMDV variants.

To select a thermostable mutant, FMDV serotype O was incubated for 30 or 60 min at 1°C below complete inactivation temperature, followed by passaging 4 to 5 generations at 37°C until the virus titers recovered, then 10 repeated cycles of thermal selection were performed, and the virus titer of each cycle of selection was determined by plaque assays. To obtain a more heat-resistant virus, the virus that passed the first round of selection was subjected to another round of selection at 2°C above the initial temperature according to the above method. Finally, the heat-resistant viruses were purified using plaque assay.

### DNA sequencing and genetic stability of thermostable FMDV variants.

The total RNA of serially passaged mutants was extracted with TRIzol reagent (Invitrogen), and the cDNA of capsid coding regions was amplified by PrimeScript reverse transcriptase (RT) master mix (TaKaRa, Dalian, China) (43) using primers 5′ GGAATGGAAAGCAAGGGTTCAGAG/ACTGGGCAAGACCTGCAAGGAA-3′ (upper) and 5′-ATGTGACCACTAAGACGGATTC/ACATGTCCTCTTGCATCTGGT-3′ (lower). The PCR product was gel purified and sequenced. Sequence alignment of the FMDV structural protein P1 was analyzed with the program ESPript. Amino acid mutations were localized on the amino acid sequence of the viral protein (i.e., in A1013T, A is mutated to T at position 13 of VP1).

### Assessment of the stability of FMDV virions.

### Particle stability thermal-release assay (PaSTRy).

A particle stability thermal release assay (PaSTRy) was performed in 96-well PCR plates using an MX3005p quantitative PCR machine (Agilent, USA) as previously described (37, 44). A reaction mixture of 50 μl containing 2 μg SDG-purified 146S particles and 5 μM SYTO 9 green fluorescent nucleic acid dye (Invitrogen) and PaSTRy buffer (2 mM HEPES and 200 mM NaCl [pH 8.0]) was run. The temperature was ramped from 25°C to 95°C in 0.5°C increments with intervals of 10 s, and the release of RNA from capsids was detected by an increase in the fluorescence signal. The minimum of the negative first derivative of the fluorescence curve was taken as the melting temperature.

### Heat resistance of inactivated virions.

To evaluate the particle stability of the mutant viruses, SDG-purified 146S particles were continuously heat dissociated at 37°C, and samples were collected every 12 h to detect the remaining intact antigen by double antibody sandwich ELISA (28, 45). Briefly, 96-well ELISA plates were coated with 0.5 μg M170 in 50 mM bicarbonate buffer (pH 9.6) at 4°C overnight and then blocked with 1% bovine serum albumin (BSA) at 37°C for 1 h. After washes, the serially diluted samples were added to the plate and incubated at 37°C for 1 h. The plates were then incubated successively with guinea pig anti-FMDV serum and horseradish peroxidase (HRP)-conjugated rabbit anti-guinea pig immunoglobulin G (IgG) at 37°C for 30 min. Finally, the plates were washed and stained with TMB for 15 min before stopping with 2 M H_2_SO_4_, and the absorbance value was measured at 450 nm. The relative antigen quantity of samples was determined based on a standard curve generated with a known FMDV virion concentration. The percentage of intact capsids to the untreated capsids was then calculated.

### Proliferation dynamics of the thermostable FMDV variants.

The rate of inactivation of wild-type and mutant viruses was determined after incubation at either 4°C for 18 days, 25°C for 60 h, or at 37°C for 24 h. In each case, virus stocks were sampled at regular time points, and the number of infectious particles was determined by plaque assay. The inactivation rate of virus infectivity was calculated by linear curve fitting of the log of the virus titer against incubation time. Along with the exponential decline of the percentage of remaining infectious particles, the dissociation rate constant was also calculated according to the following formula: *C_t_* = *C*_0_ × e^−^*^Kt^* where *t* is time, *C_t_* is virus titer at time *t*, *C*_0_ is virus titer at time zero, and *K* is the dissociation rate constant. The ratio of the *K* values of the mutant and wild-type virus was determined (21).

### Preparation of thermostable virus-like particles (VLPs).

Genes encoding VP0, VP1_A1013T_, and VP3 were fused and coexpressed, and capsid proteins were purified with His_(6)_-SUMO tag from Escherichia coli cells (46). The His_(6)_-SUMO tag was then removed from target proteins using SUMO protease. Proteins were dialyzed against assembly buffer (20 mM Tris-Cl, 500 mM NaCl, 1 mM EDTA, 1 mM dithiothreitol [DTT], and 0.1% Triton X-100 [pH 8.0]). Assembled VLPs were initially concentrated and purified by centrifugation through a 15% sucrose layer on a 60% sucrose cushion at 35,000 rpm for 3 h and further purified by centrifugation on a 7.5% to 30% sucrose density gradient at 35,000 rpm for 3 h. Fractions were all analyzed by Western blotting, and peak fraction samples were incubated at 25°C; the percentage of remaining intact capsids after incubation for a different time was further analyzed by sandwich ELISA.

### Animal experiments.

Healthy female guinea pigs were obtained from the Laboratory Animal Center of Lanzhou Veterinary Research Institute in China. Animal experiments were carried out in following the regulations for the administration of affairs concerning experimental animals approved by the State Science and Technology Commission of the People’s Republic of China and by the Committee for Animal Welfare and Safety in the Lanzhou Veterinary Research Institute of the Chinese Academy of Agricultural Sciences (no. LVRIAEC2018-008).

Guinea pigs (400 to 500 g) were randomly designated into 10 groups, with 6 guinea pigs for each experimental group that were intramuscularly vaccinated with BEI inactivated wild-type virus or mutants M3 and M10, which were all either heated for 12 h or 24 h at 37°C. Serum samples were collected at 0, 7, 14, 21, and 28 days postvaccination (dpv). Guinea pigs were subcutaneously and intradermally challenged with 0.2 ml 100 ID_50_ of a live virus on the left hind toes at 28 dpv, and clinical symptoms were observed for 7 days (46).

### Quantification of FMDV-specific antibodies.

The titer of serotype O FMDV-specific antibodies of guinea pigs sera was detected by sandwich ELISA. In brief, 96-well ELISA plates were coated with 1:1,000 diluted rabbit-anti FMDV polyclonal antibodies in 50 mM bicarbonate buffer (pH 9.6) at 4°C overnight, washed 3 times with PBS with 0.05% Tween 20 (PBST), and blocked with 1% BSA at 37°C for 1 h. Next, the plates were incubated with inactivated serotype O FMDV (from ELISA kit) at 37°C for 1 h. After the washes, 1:100-diluted serum samples were added to the plate and incubated at 37°C for 1 h, washed, and incubated with HRP-conjugated rabbit anti-guinea pig IgG at 37°C for 30 min. Finally, the plates were washed and then stained with TMB for 15 min before stopping with 2 M H_2_SO_4_ and measuring the absorbance value at 450 nm. The serotype O FMDV-positive and -negative sera were used controls.

### Quantification of FMDV neutralizing antibodies with microplate neutralizing assay.

The serum samples were heat inactivated at 56°C for 30 min, subjected to serial 2-fold dilutions in DMEM, and incubated with 100 50% tissue culture infective dose (TCID_50_) of serotype O FMDV at 37°C for 1 h in a 5% CO_2_ incubator. Next, the mixture was inoculated onto approximately 80% confluent monolayers of BHK-21 cells in 96-well cell culture plates and incubated at 37°C for 1 h. After removing the virus-serum mixture, the plate was washed twice with PBS and incubated at 37°C for 72 h. The cytopathic effect (CPE) was examined and recorded, and the titer of neutralizing antibodies was determined by the Reed-Muench formula based on 50% CPE inhibition by the diluted serum from guinea pigs.

### Data collection of cryo-EM.

Purified inactivated 146S particles (3 μl) were adsorbed on a freshly glow-discharged 400-mesh holey carbon-coated copper grid (C-flat, CF-2/1-2C; Protochips). Grids were blotted for 3 s in 90% relative humidity for plunge freezing (Vitrobot; FEI) in liquid ethane. Cryo-EM data sets were collected using a Titan Krios microscope (FEI) equipped with a direct electron detector (K2 Summit; Gatan). Movies (25 frames, each 0.2 s; total dose, 30 e^−^ Å^−2^) were recorded with a defocus between 1.2 and 2.8 μm using serial EM (47).

### Three-dimensional reconstruction and refinement.

A total of 298 micrographs (mutant M3) or 1,043 micrographs (wt virus) were recorded. Frames were corrected for a beam-induced draft by aligning and averaging each movie’s frames using MotionCorr2 (48). The contrast transfer function parameters were estimated by Gctf (49). Particles were picked manually by manual pick in RELION3 (50). A total of 3,692 (for mutant M3) and 4,168 (wt virus) particles were picked and selected for two-dimensional alignment and three-dimensional reconstruction. Finally, 3,657 (M3 mutant) and 4,045 (wt virus) particles were used for the icosahedral symmetry reconstruction. The resolution of the final icosahedral reconstructions was evaluated by Fourier shell correction (threshold, 0.143 criterion). The atomic model of FMDV (PDB: 5DJJ) was initially fitted into our maps with Chimera (51) and future corrected manually by real-space refinement in Coot (52). These models were further refined by positional and B-factor refinement in real space with phenix (53, 54). Refinement statistics are summarized in Table 2.

**TABLE 2:** Cryo-EM imaging, data processing, and model refinement statistics

Parameter	Data for:	
	Wild-type virus	Mutant M3
Data collection		
Micrographs (total)	1,043	298
Micrographs (used)	912	298
Particles selected	4,168	3,692
Particles included in final reconstruction	4,045	3,692
Sampling, Å per pixel	1.34	1.36
Defocus range (μm)/CTFFIND	1.2–3.3	1.0–2.9
Symmetry	I3	I3
Resolution (Å) (FSC = 0.5 criterion)	4	3.3
Resolution (Å) (FSC = 0.143 criterion)	3.4	3.1
Model refinement		
Ramachandran statistics (%)		
Most favored	90.51	90.65
Allowed	9.19	9.35
Outliers	0.31	0.00
RMSD^*a*^		
Bond lengths (Å)	0.005	0.006
Bond angles (°)	0.646	0.710

a RMSD, root mean square deviation.

### Multiple sequence alignment.

To analyze the conservation of mutation sites in FMDV by sequence alignment, the sequences of 43 strains of O-type FMDV and 9 strains from other serotypes (A, C, AsiaI, and SAT serotype) were download from PubMed. WebLogo generates sequence logos used with a more detailed description of sequence similarity and rapidly reveals significant features of the alignment (http://weblogo.berkeley.edu/logo.cgi) (55, 56).

### Electronic microscopy.

A total of 5 μl of each sample was adsorbed onto a carbon-coated copper grid (Pelco, CA, USA) and negatively stained with 1% (wt/vol) uranyl acetate (pH 7.4) at room temperature for 1 min. The grid was examined by transmission electron microscopy (TEM; model H-7100FA; Hitachi, Japan).

### Statistical analysis.

Statistical analyses were conducted by the two-way analysis of variance (ANOVA) method using Prism version 6.02 (GraphPad Software, La Jolla CA) for multiple comparisons. *P* values of <0.05 indicate significant differences. All error bars represent the standard error of the mean.

### Data availability.

The cryo-EM density maps of icosahedral reconstructions for FMDV serotype O and FMDV mutant M3 have been deposited in the Electron Microscopy Data Bank (https://www.ebi.ac.uk/pdbe/emdb/) under accession codes EMD-31219 and EMD-31218, respectively. The corresponding atomic coordinates have been submitted to the Protein Data Bank with accession numbers 7ENP and 7ENO.
